# The Plateletcrit and Hematocrit Levels in Premature Infants with Intraventricular Hemorrhage

**DOI:** 10.12669/pjms.41.8.11340

**Published:** 2025-08

**Authors:** Salih Cagrı Cakır, Funda Aydemir, Hilal Ozkan, Bayram Ali Dorum, Nilgun Koksal

**Affiliations:** 1 Salih Cagrı Cakır Associate Professor, Division of Neonatology, Department of Pediatrics, Bursa Uludag University Faculty of Medicine, Bursa, Turkiye; 2 Funda Aydemir, MD Division of Neonatology, Department of Pediatrics, Bursa Uludag University Faculty of Medicine, Bursa, Turkiye; 3 Hilal Ozkan Professor, Division of Neonatology, Department of Pediatrics, Bursa Uludag University Faculty of Medicine, Bursa, Turkiye; 4 Bayram Ali Dorum Associate Professor, Division of Neonatology, Department of Pediatrics, Bursa City Hospital, Bursa, Turkiye.; 5 Nilgun Koksal Professor, Division of Neonatology, Department of Pediatrics, Bursa Uludag University Faculty of Medicine, Bursa, Turkiye

**Keywords:** Intraventricular hemorrhage, Newborn, Plateletcrit, Premature infant

## Abstract

**Objective:**

We investigated the relationship between hematocrit (Hct) and plateletcrit (Pct) values and intraventricular hemorrhage.

**Methods:**

This retrospectively analyzed study conducted at the tertiary NICU (Bursa Uludag University Faculty of Medicine) between January 2016 and September 2019. The data of neonates with a gestational age of <32 weeks without and with intraventricular hemorrhage in the first three postnatal days was compared as retrospective.

**Results:**

The data of 200 patients were analyzed, including 48 patients with intraventricular hemorrhage and 152 without intraventricular hemorrhage. In the intraventricular hemorrhage group, the values were found to be significantly lower than in the non-intraventricular hemorrhage group for second-day Hct (42.9 ± 7.6 vs. 47 ± 6.6, respectively, p<0.001) and third day Hct (37.9 ± 8 vs. 43 ± 6.8, respectively, p<0.001). The third-day Pct values were lower in patients with severe intraventricular hemorrhage than in those without intraventricular hemorrhage (0.12 [0.07-0.14] vs. 0.17 [0.12-0.23], respectively, p=0.022). When the Hct and Pct values were evaluated together, it was observed that the second-day Hct×Pct values of those with second day intraventricular hemorrhage as well as the third day Hct×Pct values of those with third day intraventricular hemorrhage were lower than in patients without intraventricular hemorrhage.

**Conclusions:**

It was observed that the Hct×Pct values of the same day were lower in patients with intraventricular hemorrhage on days two and three. It was observed that there may be a relationship between low Pct and the severity of intraventricular hemorrhage. Considering the effect of erythrocyte mass on platelets, it may be more appropriate to evaluate Hct and Pct values together in considering the etiology of intraventricular hemorrhage.

## INTRODUCTION

Although survival rates in premature infants have increased in parallel with developments in neonatal intensive care units (NICUs), the frequency of intraventricular hemorrhage (IVH) remains high.^1^ The risk of IVH in premature infants (15%-40%) increases inversely with the gestational age.^1^,^2^ The first week of life is the riskiest period, and approximately 90% of IVHs occur within the first 72 hours.^1^,^2^ The pathophysiology of IVH is multifactorial in premature infants, with the main causes being high vascularity in the germinal matrix, insufficient vascular bed support, variability in cerebral blood flow, and coagulation/platelet disorders.^2^,^3^ To date, however, studies have not found a determinant parameter for the increased risk of IVH.^1^

A better understanding of the factors responsible for the development of IVH will enable the development of neuroprotective strategies. Platelet mass is as important as platelet count in platelets’ functioning.^2^,^4^ In addition, it is believed that low blood volume may increase the cerebral blood flow, promoting a tendency to IVH.^5^ Physiologically, the greatly larger erythrocytes flow in the middle of the vessel and the platelets flow close to the vessel wall during blood flow in the vascular bed, and this arrangement plays a positive auxiliary factor role in the functioning of platelets.^6^ Thus, erythrocyte mass is also important in the functioning of platelets. This study investigated the relationship between erythrocyte percentage (hematocrit [Hct]), platelet percentage (plateletcrit [Pct]), and IVH development.

## METHODS

The study’s researchers retrospectively analyzed the electronic files of infants born at less than 32 weeks of gestation who were hospitalized in the tertiary NICU (Bursa Uludag University Faculty of Medicine) between January 2016 and September 2019. Patients who were admitted to the unit more than 24 hours after birth, had congenital anomalies incompatible with life, and whose postpartum cranial ultrasonography, complete blood count, and coagulation parameters were not evaluated were excluded from the study. Patients with IVH detected after the third day of life were excluded from the evaluation.

### Ethical Approval:

The study was granted ethics committee approval (2019-14/40; dated September 9, 2019).

### Patient data and characteristics:

The researchers recorded gestational week, birth weight, Apgar scores, gender, delivery type, cord clamping times, antenatal steroid application, maternal diabetes, hypertension, presence of hemodynamically significant patent ductus arteriosus (HsPDA), mechanical ventilator requirement and method in the first 72 hours, surfactant need, surfactant administration routes, sepsis situations, need for transport from an external center, cranial ultrasonography findings and presence of pneumothorax in the first 72 hours after birth, complete blood count, activated partial thromboplastin time (APTT), prothrombin time (PT) measurement results, and erythrocyte transfusion information.

Following our NICU practices, complete blood counts and cranial ultrasonography are scanned from high-risk premature infants on the first day, second day and third day after birth. In addition, coagulation parameters are cheked in these infants during their initial admission. Cranial ultrasound timing is approximately 6-24 hours for the first day, 36-48 hours for the second day, and 60-72 hours for the third day. The timing to have a complete blood count is approximately 0-2 hours for the first day, 24-36 hours for the second day, and 48-72 hours for the third day. These data were obtained from the records of the study patients. The IVH grades were classified as defined in the literature.^5^ The presence of coagulopathy was evaluated according to the patients’ PT and APTT reference values.^7^ Patients with IVH in the first three postnatal days and without IVH were compared.

### Definitions

In the NICU, erythrocyte transfusions are performed according to the threshold hemoglobin values reported by the Turkish Neonatology Society.^8^ Echocardiography screening and treatment of premature infants for HsPDA are performed according to the criteria recommended by the Turkish Neonatology Society.^9^ Pct represents the volume of platelets in the blood by percentage and is calculated by multiplying the mean platelet volume in a femtoliter by the platelet count (1,000/uL) and dividing by 10,000.^10^

This study investigated the relationship between the patients’ demographic and clinical characteristics; the platelet count, Pct, Hct, and coagulation values; and the presence of IVH. The data of patients with and without IVH were compared. As there is no separately defined formula for evaluating the effect of erythrocyte mass on Pct, this situation was evaluated with the Hct×Pct result. Hypercapnia and hypocapnia were evaluated when PaCO_2_ level was > 55 mmHg and < 35 mmHg in blood gas analyses, respectively.^11^ Hypoglycemia and hyperglycemia were defined when the blood glucose level was < 47 mg/dl and > 180 mg/dl, respectively.^12^,^13^

### Statistical analysis:

The data were evaluated by descriptive statistical methods and the findings were analyzed using SPSS version 25. The chi-square test was used for categorical variables. The distribution of numerical variables was tested with the Shapiro-Wilk test, and those with normal distribution were compared with the t-test, whereas those not exhibiting normal distribution were compared with the Mann-Whitney U test. Logistic regression analysis was applied for variables that showed statistically significant differences between the groups with and without IVH. *P*-values of <0.05 were considered statistically significant.

## RESULTS

**Fig.1:**
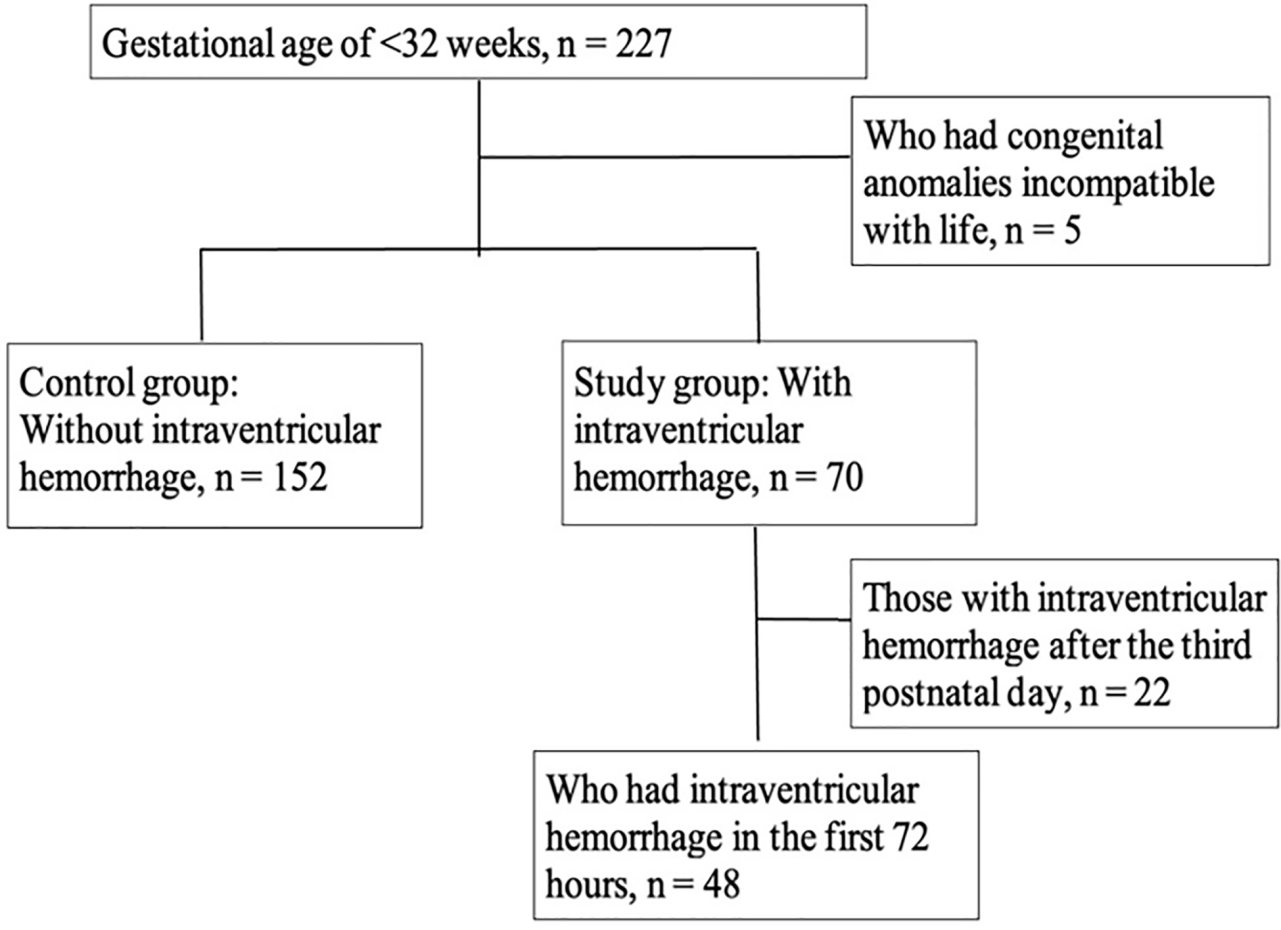
The study flowchart.

The data of 48 patients with IVH and 152 premature infants without IVH were analyzed. Fig.1 shows the patient enrolment flow chart. Among neonates with IVH, the numbers at Grades-1, 2, and 3 and with periventricular hemorrhagic infarcts (PVHIs) were 18 (37.5%), 22 (45.8%), 2 (4.2%), and 6 (12.5%), respectively. In the follow-up, one patient needed a lumbar puncture, and one patient needed a reservoir, but no patient needed a ventriculoperitoneal shunt.

The mean gestational week and birth weight of the IVH group were lower than in the non-IVH group. The HsPDA rates, mortality rates, and numbers of erythrocyte transfusions, intubated mechanical ventilator support, surfactant requirements, normal vaginal deliveries, and pneumothorax were statistically significantly higher in the IVH group. The demographic and clinical characteristics of the neonates in both groups are given in Table-I.

**Table-I:** Demographic and clinical characteristics of the neonates.

	With IVH, (n=48)	Without IVH, (n=152)	p
Gestational age (weeks), mean ± SD	28.3 ± 2.1	29.7 ± 2	***<0.001 ^a^
Birth weight (g), mean ± SD	1165 ± 381	1368 ± 467	**0.007 ^a^
Vaginal birth, n (%)	17 (35.5)	17 (11.2)	***<0.001 ^b^
Maternal diabetes, n (%)	5 (10.4)	12 (7.9)	0.585 ^b^
Maternal hypertension, n (%)	12 (25)	38 (25)	1^b^
Maternal smoking, n (%)	2 (4.1)	4 (2.6)	NA^b^
Chorioamnionitis, n (%)	11 (23)	29 (19)	0.562 ^b^
Gender (male), n (%)	31 (64.5)	90 (59)	0.507 ^b^
1. minute Apgar score, mean ± SD	5.5 ± 2	5.6 ± 2.3	0.653 ^a^
5. minute Apgar score, mean ± SD	7.1 ± 1.9	7.4 ± 1.8	0.395 ^a^
Delivery room resuscitation			
Need for positive pressure ventilation, n (%)	7 (14.5)	19 (12.5)	0.708^b^
Need for intubation, n (%)	13 (27)	36 (23.6)	0.633^b^
Need for chest compressions, n (%)	0	4 (2.6)	NA ^b^
Antenatal steroid administration, n (%)	29 (60)	98 (64.4)	0.632 ^b^
Delayed cord clamping, n (%)	6 (12.5)	18 (11.8)	0.903 ^b^
Transport, n (%)	7 (14.5)	17 (11)	0.528 ^b^
Need for invasive mechanical ventilation, n (%)	38 (79)	78 (51.3)	***0.001^b^
Need surfactant therapy, n (%)	35 (73)	81 (53.2)	*0.016 ^b^
Surfactant administration via an endotracheal tube, n (%)	33 (68.7)	70 (46)	0.218^b^
Hemodynamically significant patent ductus arteriosus, n (%)	31 (64.5)	66 (43.4)	**0.007^b^
Need for erythrocyte transfusions in the first 72 hours, n (%)	11 (23)	11 (7.2)	**0.002^b^
Pneumothorax, n (%)	5 (10)	5 (3.2)	*0.048^b^
Hypoglycemia, n (%)	8 (16.6)	20 (13)	0.541 ^b^
Hyperglycemia, n (%)	2 (4.1)	11 (7.2)	0.452 ^b^
Hypocapnia, n (%)	4 (8.3)	18 (11.8)	0.498 ^b^
Hypercapnia, n (%)	14 (29)	29 (19)	0.138 ^b^
Mortality, n (%)	16 (33.3)	28 (18.4)	*0.03^b^

a: student t test, b: Chi-square test, IVH: intraventricular hemorrhage, SD: standard deviation.

On the second and third days, the Hct and Hct×Pct values of patients with IVH were found to be statistically significantly lower. The laboratory characteristics of the neonates in both groups on the first, second, and third day as well as the presence of coagulopathy according to the PT and APTT values are given in Table-II.

**Table-II:** Laboratory characteristics of the neonates.

		With IVH (n=48)	Without IVH (n=152)	p
Hematocrit (%)	1^st^-day	47.2 ± 7	49.4 ± 7.1	0.072^a^
	2^nd^-day	42.9 ± 7.6	47.0 ± 6.6	***<0.001^a^
	3^rd^-day	37.9 ± 8	43.3 ± 6.8	***<0.001^a^
Platelet count (10^9^/L)	1^st^-day	201 ± 76	208 ± 73	0.591^a^
	2^nd^-day	168 ± 70	185 ± 75	0.162^a^
	3^rd^-day	172 ± 79	200 ± 110	0.101^a^
Plateletcrit (%)	1^st^-day	0.16 ± 0.06	0.16 ± 0.057	0.949^a^
	2^nd^-day	0.14 ± 0.056	0.15 ± 0.054	0.304^a^
	3^rd^-day	0.15 ± 0.074	0.18 ± 0.08	0.098 ^a^
Hematocrit x Plateletcrit	1^st^-day	7.5 ± 2.8	7.9 ± 2.8	0.506^a^
	2^nd^-day	6 ± 2.3	7.0 ± 2.5	*0.034^a^
	3^rd^-day	6 ± 2.8	7.9 ± 3.5	**0.003^a^
Coagulopathy according to PT, n (%)	1^st^-day	32 (66)	83 (54.6)	0.141^b^
Coagulopathy according to APTT, n (%)	1^st^-day	9 (18.7)	17 (11.1)	0.174^b^

a: student t test, the data are shown as mean ± standard deviation, b: Chi-square test, IVH: intraventricular hemorrhage, PT: prothrombin time, APTT: activated partial thromboplastin time.

Table-III shows the evaluation of the hematological findings of the patients with IVH according to the hemorrhage day as well as their statistical comparison with the non-IVH patients. The Hct levels of patients with IVH on the second and third days were lower. In addition, the Hct×Pct values on the second day of the patients with IVH on the second day and the Hct×Pct values on the third day of the patients with IVH on the third day were statistically significantly lower.

**Table-III:** The evaluation of the hematological findings of the patients according to the hemorrhage day.

		Without IVH (n=152)	The patients with IVH according to the hemorrhage day (n=48)					
			1^st^-day, (n=21)		2^nd^-day, (n=16)		3^rd^-day, (n=11)	
Hematocrit (%)	1^st^-day	49.5 (45-53)	48.2 (41-52)	0.210^a^	48.6 (44-50)	0.306^b^	46.9 (46-52)	0.441^c^
	2^nd^-day	46.4 (43-51)	46.1 (41-49)	0.260^a^	43.3 (35-48)	*0.018^b^	42.4 (35-46)	*0.01^c^
	3^rd^-day	42.9 (38-47)	40.7 (31-43)	*0.019^a^	40.6 (32-46)	*0.043^b^	35.8 (30-39)	***<0.001^c^
Plateletcrit (%)	1^st^-day	0.15 (0.11-0.19)	0.15 (0.11-0.20)	0.516^a^	0.13 (0.12-0.17)	0.483^b^	0.14 (0.10-0.17)	0.382^c^
	2^nd^-day	0.15 (0.10-0.18)	0.14 (0.11-0.18)	0.842^a^	0.11 (0.08-0.16)	0.103^b^	0.13 (0.10-0.18)	0.800^c^
	3^rd^-day	0.17 (0.12-0.23)	0.14 (0.11-0.19)	0.415^a^	0.12 (0.10-0.23)	0.261^b^	0.12 (0.07-0.19)	0.109^c^
Hematocrit x Plateletcrit	1^st^-day	7.5 (6.0-9.6)	8.0 (5.6-9.7)	0.800^a^	6.5 (4.7-9.8)	0.404^b^	7.2(5.1-8.5)	0.450^c^
	2^nd^-day	6.9 (5.0-8.9)	6.4 (5.3-7.7)	0.277^a^	5.3 (3.4-7.9)	*0.029^b^	5.6 (4.7-8.1)	0.567^c^
	3^rd^-day	7.5 (5.3-9.9)	5.7 (4.9-7.6)	0.055^a^	5.5 (3.8-9.6)	0.097^b^	4.3 (2.1-7.9)	*0.023^c^

p: Mann Whitney U test, the data are shown as median and interquartile range, a: Comparison of those with IVH on the 1st-day and those without IVH, b: Comparison of those with IVH on the 2nd-day and those without IVH, c: Comparison of those with IVH on the 3rd-day and those without IVH, IVH: intraventricular hemorrhage.

Table-IV shows the evaluation of the hematological findings of patients with mild (Grades-1 and 2) and severe (grade 3 and PVHI) IVH and their statistical comparison with patients without hemorrhage. It was observed that, on the third day, the average Pct value of the patient group with severe IVH was significantly lower than that of those without IVH. Logistic regression analysis was performed for the variables in which statistically significant differences were observed between the groups with and without hemorrhage. The analysis revealed that normal vaginal delivery was higher in the IVH group (Odds Ratio: 3.922, *P*: 0.015, CI %95: 1.309-11.752).

**Table-IV:** Hematological characteristics of patients according to the IVH grade.

		Without IVH (n=152)	The patients with IVH according to the hemorrhage grade (n=48)			
			Mild (grade 1 and 2) (n=40), p		Severe (grade 3 and PVHI) (n=8), p	
Hematocrit (%)	1^st^-day	49.5 (45-53)	48.1 (45-50)	0.159^a^	46.4 (39-54)	0.291^b^
	2^nd^-day	46.4 (43-51)	44.4 (39-48)	*0.021^a^	38.4 (32-46)	0.007^b^
	3^rd^-day	42.9 (38-47)	40.6 (31-43)	**0.001^a^	35.6 (26-39)	**0.001^b^
Plateletcrit (%)	1^st^-day	0.15 (0.11-0.19)	0.14 (0.11-0.19)	0.785^a^	0.15 (0.10-0.19)	0.865 ^b^
	2^nd^-day	0.15 (0.10-0.18)	0.13 (0.10-0.17)	0.226^a^	0.13 (0.11-0.19)	0.826^b^
	3^rd^-day	0.17 (0.12-0.23)	0.14 (0.10-0.22)	0.278^a^	0.12 (0.07-0.14)	*0.022^b^
Hematocrit x Plateletcrit	1^st^-day	7.5 (6.0-9.6)	7.4 (5.5-9.5)	0.644^a^	7.1 (4.9-9.7)	0.647^b^
	2^nd^-day	6.9 (5.0-8.9)	5.9 (4.0-8.0)	0.08^a^	5.2 (4.7-6.5)	0.132^b^
	3^rd^-day	7.5 (5.3-9.9)	5.7 (4.4-8.3)	*0.028^a^	4.0 (1.2-5.3)	**0.003^b^

p: Mann Whitney U test, the data are shown as median and interquartile range, a: Comparison of those with mild IVH and those without IVH, b: Comparison of those with severe IVH and those without IVH, IVH: intraventricular hemorrhage, PVHI: periventricular hemorrhagic infarction.

## DISCUSSION

This study compared the perinatal characteristics, neonatal characteristics, and laboratory findings of premature infants with and without IVH who had a gestational age of <32 weeks. The Hct and Pct values were evaluated separately and together, and their relationships to the development of IVH were examined. A significant decrease in Hct and Hct×Pct was found on the second and third days in IVH patients. In addition, when the patients were divided into groups and their hematological results were examined according to hemorrhage day, we observed that the Hct×Pct values were lower only on second day in those with hemorrhage on the second day and only on third day in those with hemorrhage on the third day. In this study, the analysis together of repeated hematological markers and the presence of IVH during the first three postnatal days were strengthened the assessment of time-related correlation. To our knowledge, this study is among the first to examine the combined assessment of Hct and Ptc in relation to IVH in preterm infants. In clinical follow-up, HctxPct values may be helpful in assessing the risk of IVH. In addition, the association between severe IVH and lower Pct levels may be helpful in predicting the severity of IVH.

In our study, infants who developed IVH required a significantly higher rate of erythrocyte suspension transfusion in the first 72 hours. Christensen et al.^14^ have shown that reducing transfusions in the first days of life is associated with a decrease in the development of IVH, but the causality has not been fully elucidated, as it is unclear whether the increase in IVH rate is due to the transfusion itself or to the conditions that require the transfusion. It is speculated that transfusion itself may lead to IVH by causing obstruction and injury in the choroid plexus/germinal matrix capillaries due to reactive oxygen species and endothelial activation or because of the structural features of donor erythrocytes.^14^ In addition, it has been reported that low blood volume may contribute to the development of IVH, and situations that may cause anemia should be avoided.^14^ In our study, the second and third day Hct values of IVH patients were found to be lower.

When the patients with IVH were examined according to hemorrhage day, it was observed that all the IVH patients had lower Hct values on the third day compared to the control group; those with second and third day hemorrhage also had lower Hct values on the second day. The evaluation suggests that low Hct may develop due to bleeding. In addition, it was observed that the measured Hct×Pct values were lower only on the second day in those with hemorrhage on the second day and only on the third day in those with hemorrhage on the third day. This suggests that Hct×Pct evaluation may provide a better evaluation than Hct and Pct evaluation separately, but these results were not found to be an independent risk factor.

Our study could not clearly demonstrate a causal relationship between the low Hct and increased transfusion rates observed in IVH patients and the development of IVH. However, based on the fact that low blood volume and Hct value may adversely affect platelet functioning, low Hct, Pct, and Hct×Pct values were found to be associated with IVH, and we speculate that there may be a causal relationship. Studies in the literature report that low Pct at birth is associated with an increased risk of IVH development in neonates born at <32 weeks gestation,^10^,^15^ but it has also been reported that high mean platelet volume in the first three days is associated with increased IVH in very low birth weight infants.^16^,^17^ It has also been reported that the average Pct value in premature infants is lower than in term infants.^18^ The evaluation of Pct values together with Hct may provide additional benefits in evaluating the risk of IVH in premature infants. There is a need to evaluate the relationship between the effect of erythrocyte mass on platelet functions and the development of IVH in premature infants by clinical studies.

Thrombocytopenia and coagulopathy can play a role in the etiology of IVH in term and preterm neonates.^19^ Our study included premature infants, and the platelet counts and coagulopathy rates were similar in both the IVH and non-IVH groups. However, in the development of IVH in premature infants, the fragile structure of the germinal matrix and changes in intracranial blood flow should be considered as well as coagulopathy and thrombocytopenia. IVH occurs mostly in the first three days of life,^5^,^14^ and the data of the cases in our study include the first three postnatal days. Aligning with the literature, Grade-1 and 2 hemorrhages were detected in the majority of our patients (83%) and severe hemorrhages in the remainder (17%), although the Pct levels were found to be similar in patients with and without IVH.^5^ When evaluated according to IVH grade, it was seen that the third-day Pct values of patients with severe IVH were lower than those of patients at without IVH. This suggests that Pct values may be related to IVH weight. Similarly, Hsieh et al.^10^ report that low Pct at birth is an independent risk factor for the development of severe IVH in the first seven postnatal days in neonates born at <32 weeks gestation.

Our study found that the rate of normal vaginal delivery was higher in the IVH group and was an independent risk factor for IVH. The study of Humberg et al.^20^ report that elective cesarean section in premature infants of <30 weeks is associated with a decrease in the risk of IVH. Likewise, lower rates of IVH have been reported in premature infants born at <32 weeks by cesarean section without uterine contraction.^21^ However, there is not enough evidence to support cesarean delivery as a preventive factor.^19^,^22^ An important antenatal practice that reduces the risk of IVH is antenatal steroid administration.^23^,^24^ In our study, it was found that antenatal steroid administration rates were low in both groups and did not show any statistical difference. It was thought that there was no statistical difference, unlike the literature, due to the low antenatal steroid rates of our study and the small number of patients. Hypoglycemia/hyperglycemia and hypercapnia/hypocapnia conditions may increase the risk of IVH by causing cerebral blood flow changes.^23^,^11^ In our study, the results between groups were similar. We thought this was because the study was retrospective and PaCO_2_ fluctuations could not be detected.

### Limitations

It includes its retrospective design and the fact that the cranial ultrasonography hours were not standardized. The retrospective nature of the study limits causal inferences. While the association between lower Hct×Pct and IVH is statistically significant, causality cannot be determined.

## CONCLUSION

In conclusion, in light of the independent relationship between IVH and Hct and Pct values as well as the effect of erythrocyte mass on platelets, it may be more appropriate to evaluate Hct and Pct together. Pct, which may be associated with IVH severity, is a parameter that should be considered in routine blood count follow-ups. There is a need for prospective studies that examine the relationship between Hct and Pct and the development of IVH, that standardize cranial imaging and Hct and Pct evaluation hours, and that examine more patients.

### Author’s Contributions:

**SCC, BAD, FA and NK:** Conceptualization.

**FA, BAD, and NK:** Data curation, Formal analysis, Funding acquisition.

**FA, SCC, BAD and HO:** Investigation, Methodology.

**SCC, FA, HO and SCC:** Software, Validation, Visualization.

**SCC, BAD and FA:** Writing original draft.

**HO and NK:** Writing - review & editing.

**SCC, BAD, FA and NK:** Responsible and accountable for the accuracy or integrity of the work.

## References

[1] Siddappa AM, Quiggle GM, Lock E, Rao RB (2021). Predictors of severe intraventricular hemorrhage in preterm infants under 29-weeks gestation. J Matern Fetal Neonatal Med.

[2] Μitsiakos G, Papathanasiou AE, Kyriakidis I, Karagianni P, Tsepis K, Tzimou I (2016). Intraventricular hemorrhage and platelet indices in extremely premature neonates. J Pediatr Hematol Oncol.

[3] Leijser LM, de Vries LS (2019). Preterm brain injury:Germinal matrix-intraventricular hemorrhage and post-hemorrhagic ventricular dilatation. Handb Clin Neurol.

[4] Korkmaz L, Bastug O, Ozdemir A, Ceylan M, Gunes T, Ozturk MA (2019). Can platelet mass index be a parameter to predict intraventricular hemorrhage in very-low-birth-weight newborns?. Am J Perinatol.

[5] Inder TE Perlman JM, Volpe JJ, Volpe JJ (2018). Preterm intraventricular hemorrhage/posthemorrhagic hydrocephalus. Volpe's Neurology of the Newborn.

[6] Weisel JW, Litvinov RI (2019). Red blood cells:the forgotten player in hemostasis and thrombosis. J Thromb Haemost.

[7] Andrew M, Paes B, Milner R, Johnston M, Mitchell L, Tollefsen DM (1988). Development of the human coagulation system in the healthy premature infant. Blood.

[8] Çetinkaya M, Atasay B, Perk Y (2018). Turkish Neonatal Society guideline on the transfusion principles in newborns. Turk Pediatri Ars.

[9] Köksal N, Aygün C, Uras N (2018). Turkish Neonatal Society guideline on the management of patent ductus arteriosus in preterm infants. Turk Pediatri Ars.

[10] Hsieh PY, Hsu KH, Chiang MC, Hsu JF, Chu SM, Lien R (2023). Platelet parameters and the association with morbidity and mortality in Preterm Infants. Pediatr Neonatol.

[11] Vela-Huerta MM, Amador-Licona M, Medina-Ovando N, Aldana-Valenzuela C (2009). Factors associated with early severe intraventricular haemorrhage in very low birth weight infants. Neuropediatrics.

[12] Aliefendioğlu D, Çoban A, Hatipoğlu N, Ecevit A, Arısoy AE, Yeşiltepe G (2018). Management of hypoglycemia in newborn:Turkish Neonatal and Pediatric Endocrinology and Diabetes Societies consensus report. Turk Pediatri Ars.

[13] Şimşek DG, Ecevit A, Hatipoğlu N, Çoban A, Arısoy AE, Baş F (2018). Neonatal Hyperglycemia, which threshold value, diagnostic approach and treatment?:Turkish Neonatal and Pediatric Endocrinology and Diabetes Societies consensus report. Turk Pediatri Ars.

[14] Christensen RD, Baer VL, Lambert DK, Ilstrup SJ, Eggert LD, Henry E (2014). Association, among very-low-birthweight neonates, between red blood cell transfusions in the week after birth and severe intraventricular hemorrhage. Transfusion.

[15] Go H, Ohto H, Nollet KE, Takano S, Kashiwabara N, Chishiki M (2020). Using platelet parameters to anticipate morbidity and mortality among preterm neonates:a retrospective study. Front Pediatr.

[16] Cekmez F, Tanju IA, Canpolat FE, Aydinoz S, Aydemir G, Karademir F (2013). Mean platelet volume in very preterm infants:a predictor of morbidities?. Eur Rev Med Pharmacol Sci.

[17] Varal IG, Celik EDA, Dogan P, Tunc G, Oren A (2025). The prognostic value of mean platelet volume levels in germinal matrix hemorrhage- intraventricular hemorrhage in preterm infants. Pak J Med Sci.

[18] Alva SR, Ashwini KT, Navya BN (2016). Comparative Study of Platelet Indices between Term, Preterm and Small for Gestational Age Newborns. Sch J App Med Sci.

[19] De Vries LS, Leijser LM, Martin R, Nordli DR (2023). Germinal matrix and intraventricular hemorrhage (GMH-IVH) in the newborn:Risk factors, clinical features, screening, and diagnosis. UpToDate.

[20] Humberg A, Hartel C, Paul P, Hanke K, Bossung V, Hartz A (2017). Delivery mode and intraventricular hemorrhage risk in very-low-birth-weight infants:Observational data of the German Neonatal Network. Eur J Obstet Gynecol Reprod Biol.

[21] Poryo M, Boeckh JC, Gortner L, Zemlin M, Duppre P, Ebrahimi Fakhari D (2018). Ante-, peri- and postnatal factors associated with intraventricular hemorrhage in very premature infants. Early Hum Dev.

[22] Haque KN, Hayes AM, Ahmed Z, Wilde R, Fong CY (2008). Caesarean or vaginal delivery for preterm very-low-birth weight (<or =1,250 g) infant:experience from a district general hospital in UK. Arch Gynecol Obstet.

[23] Çizmeci MN, Akın MA, Ozek E (2021). Turkish Neonatal Society Guideline on the Diagnosis and Management of Germinal Matrix Hemorrhage-Intraventricular Hemorrhage and Related Complications. Turk Arch Pediatr.

[24] McGoldrick E, Stewart F, Parker R, Dalziel SR (2020). Antenatal corticosteroids for accelerating fetal lung maturation for women at risk of preterm birth. Cochrane Database Syst Rev.

